# Development and evaluation of a bioinformatics approach for designing molecular assays for viral detection

**DOI:** 10.1371/journal.pone.0178195

**Published:** 2017-05-25

**Authors:** Pierre H. H. Schneeberger, Joël F. Pothier, Andreas Bühlmann, Brion Duffy, Christian Beuret, Jürg Utzinger, Jürg E. Frey

**Affiliations:** 1 Agroscope, Department of Methods Development and Analytics, Wädenswil, Switzerland; 2 Department of Virology, Spiez Laboratory, Federal Office for Civil Protection, Spiez, Switzerland; 3 Swiss Tropical and Public Health Institute, Basel, Switzerland; 4 University of Basel, Basel, Switzerland; 5 Zurich University of Applied Sciences (ZHAW), Institute of Natural Resource Sciences, Environmental Genomics and Systems Biology Research Group, Wädenswil, Switzerland; 6 Department of Foods of Plant Origin, Agroscope, Institute for Food Sciences IFS, Wädenswil, Switzerland; Metabiota, UNITED STATES

## Abstract

**Background:**

Viruses belonging to the *Flaviviridae* and *Bunyaviridae* families show considerable genetic diversity. However, this diversity is not necessarily taken into account when developing diagnostic assays, which are often based on the pairwise alignment of a limited number of sequences. Our objective was to develop and evaluate a bioinformatics workflow addressing two recurrent issues of molecular assay design: (i) the high intraspecies genetic diversity in viruses and (ii) the potential for cross-reactivity with close relatives.

**Methodology:**

The workflow developed herein was based on two consecutive BLASTn steps; the first was utilized to select highly conserved regions among the viral taxon of interest, and the second was employed to assess the degree of similarity of these highly-conserved regions to close relatives. Subsequently, the workflow was tested on a set of eight viral species, including various strains from the *Flaviviridae* and *Bunyaviridae* families.

**Principal findings:**

The genetic diversity ranges from as low as 0.45% variable sites over the complete genome of the Japanese encephalitis virus to more than 16% of variable sites on segment L of the Crimean-Congo hemorrhagic fever virus. Our proposed bioinformatics workflow allowed the selection—based on computing scores—of the best target for a diagnostic molecular assay for the eight viral species investigated.

**Conclusions/Significance:**

Our bioinformatics workflow allowed rapid selection of highly conserved and specific genomic fragments among the investigated viruses, while considering up to several hundred complete genomic sequences. The pertinence of this workflow will increase in parallel to the number of sequences made publicly available. We hypothesize that our workflow might be utilized to select diagnostic molecular markers for higher organisms with more complex genomes, provided the sequences are made available.

## Introduction

The genus *Flavivirus* (RNA virus) includes several species that cause serious human diseases. In *Flavivirus* infections, the first clinical features observed include, but are not limited to, fever, myalgia, headaches, and other nonspecific symptoms [1–4]. These nonspecific symptoms complicate the identification of the specific causative agent. Importantly, Japanese encephalitis virus (JPEV), West Nile virus (WNV), and St. Louis encephalitis virus (SLEV) are responsible for larger outbreaks affecting both humans and animals [5–7]. Other emerging zoonotic *Flaviviruses*, such as the Usutu virus (USUV), might become important threats to human health due to their similarities with other human pathogenic viruses, such as WNV [8, 9]. While potential vectors are expanding in the northern hemisphere, resulting in sporadic cases of WNV [10, 11] and USUV infections in birds [12, 13], these infections remain endemic in low- and middle-income countries. New research is needed to develop methods for rapid and accurate identification, and to validate these diagnostic tests before wider application. Additionally, while other zoonotic arboviruses, such as the Rift Valley fever virus (RVFV) and the Crimean-Congo hemorrhagic fever virus (CCHFV) within the *Bunyaviridae* family, cause serious diseases in humans, only a limited number of assays are currently available for their identification and there is a lack of standardization in the assays used in routine diagnostics laboratories [14, 15].

Virus neutralization tests (VNTs) are usually considered the ‘gold’ standard for the diagnosis of infections by these pathogens [16]. VNTs, however, require a cultivation step that must be performed in laboratories with high biosafety measures, which are not widely available in low- or middle-income countries. Immunoassays are broadly used in clinical-diagnostic settings. However, while immunoassays rely on biochemistry to identify the presence or concentration of antibodies or antigens, genomic and phylogenetic information to understand the route of transmission and biology of these viruses is lacking. Various polymerase chain reaction (PCR)-based assays, including real-time PCR, have been used successfully in epidemiologic studies [17–19]. Yet, this variety of assays introduces a lack of standardization in the different routine diagnostic laboratories. It is conceivable that taxon-specific molecular assays, even though system-wide diagnostics studies become more and more common [20], that are relying on genomic information might help clinicians and researchers to obtain more accurate epidemiologic baseline data for neglected viral infections [21–23]. Within the *Bunyaviridae* family, viruses from the *Hantavirus* genus are responsible for several recent outbreaks [24–26], but reliable molecular assays to trace transmission pathways and to deepen our understanding of viral epidemiology have yet to be developed and more widely implemented.

Genetic diversity among RNA viruses from the *Bunyaviridae* and *Flaviviridae* families is high compared with that of DNA viruses, as has been shown by new data produced by next-generation sequencing technologies [27, 28]. While the development of molecular assays is quite straightforward, such approaches are mainly based on the pairwise alignments of sequences, followed by selection of the most conserved region within the aligned sequences. Although alignment algorithms are constantly being improved, computational challenges are still encountered when dealing with large numbers of sequences. Such molecular assays are of low priority for organisms with slow mutation rates because the overall genetic diversity of these organisms remains low and few sequences are sufficient to create an accurate representation. In contrast, in rapidly mutating viruses, the method may become restrictive because of the small number of sequences, which may not necessarily represent the complete genetic diversity within the species. Thus, overall, this alignment approach may give rise to two challenges: (i) the selected region is only conserved among a few genetic variants and not among the complete taxon and (ii) lack of information about the degree of sharing between the selected regions and the sequences of other closely related organisms, potentially causing cross-reactions.

We developed a workflow based on the well-established BLASTn algorithm [29] to address the aforementioned challenges. Subsequently, the workflow was tested on a set of viruses from the *Flaviviridae* and *Bunyaviridae* families. Our data may be applicable for rapid selection of highly conserved and taxon-specific regions for any viral family and, perhaps, for other higher organism for which sufficient genomic data are available. This may further improve various nucleic acid-based molecular tools, such as real-time PCR or loop-mediated isothermal amplification (LAMP).

## Methods

### Hardware and software requirements

Version 2.2.28+ (64 bits) of the standalone BLAST algorithm was employed in the workflow. A backbone script written in PERL was utilized to automate the process and to parse and retrieve the intermediate and final result files. The workflow was tested on two versions of PERL (versions 5.16 x64 and 5.10 x32). Of note, the script will work with any other PERL version compatible with the BioPerl package v.1.6.901 [30]. Version 2.3.4 of the Primer3 package [31] was utilized to select primers for the real-time PCR assays. For each species, a subset of highly conserved fragments (HCFs; *n* = 2) selected by the workflow was used to design a primer pair for real-time PCR analysis. In order to test different assay configurations, we used the “pick primers tool” from Primer3 with a primer size range set to 18–24-mer primers, and a target amplification product size set between 300 and 400 bp for members of the *Flaviviridae* family. The same “pick primers tool” was used for members of the *Bunyaviridae* family; however, because of the higher genetic variability, the primer size range was adjusted to generate 25–30-mer primers, and the amplification product target size was set between 100 and 400 bp.

The same sets of HCFs selected for real-time PCR assays were used as the amplification target to test LAMP assays. The HCFs for SLEV and USUV were submitted to the online LAMP primer design tool Primer Explorer V4 (Fujitsu, Japan; see: https://primerexplorer.jp/). A set of six LAMP primers (F3, B3, FIP, BIP, LoopF, and LoopB) was automatically selected for each of the two species.

To demonstrate the flexibility of this workflow, two different computer configurations were used. Configuration “1” was a conventional notebook, running Windows 7 (x64) with 8 Gigabyte (Gb) of RAM and an i7 quad core CPU to run up to eight BLASTn instances in parallel. Configuration “2” was a more powerful workstation running Windows 7 (x64), with 32 Gb of RAM and an i7 hexacore CPU able to run up to 12 BLASTn instances in parallel.

### Input data used for the workflow

A file containing all publicly available complete genome sequences was downloaded on January 17, 2013 for each tested virus species from GenBank [32]. The number of sequences available on this date ranged from only six sequences for USUV up to 608 sequences for WNV (S1 Table).

### Phylogenetic analyses

Phylogenetic analysis was performed using MEGA v.6.0 software [33]. The ClustalW pairwise alignment algorithm [34] was used with default parameters, and the trees were generated from the sequence alignments using the neighbor-joining approach [35] with 700 bootstrap replications.

### Viral samples

Eight viral species from the *Flaviviridae* and the *Bunyaviridae* families were used to test the results of the workflow. Two WNV strains (i.e., NY99 and Dakar) were included in this study. For the remaining seven viral species, we included a single species sample and did not test various strains. The viral samples were obtained from various European collections and cultivated using various methods, as reported in Table 1. Upon receipt, each virus was propagated in appropriate cell cultures within a biosafety level 3 (BSL-3) facility at Spiez Laboratory (Spiez, Switzerland) and virus titers were measured using the respective validated rt-qPCR protocols. An aliquot of each sample was stored at -80°C.

**Table 1:** Virus species used for the validation of the diagnostic assays developed with the workflow designed in this study.

Taxonomy (family, genus, species)			Abbreviation	Subtype	Cell type	Origin^a^
***Flaviviridae***						
	***Flavivirus***					
		St. Louis encephalitis virus	SLEV	Type 1	Vero E6-Lyon	NCPV
		Usutu virus	USUV	Bologna	Vero E6-Lyon	UNIBO
		Tick-borne encephalitis virus	TBEV	Hanzalova	Porcine kidney	IP ASCR
		Japanese encephalitis virus	JPEV	Nakayama	Vero E6-Lyon	NCPV
		West Nile virus	WNV	NY99	Vero E6-Lyon	NCPV
		West Nile virus	WNV	Dakar	Vero E6-Lyon	NCPV
***Bunyaviridae***						
	***Nairovirus***					
		Crimean-Congo hemorrhagic fever virus	CCHFV	N.A.^b^	BNI	BNI
	***Phlebovirus***					
		Rift Valley fever virus	RVFV	H13/96	Vero E6-Lyon	NCPV
	***Hantavirus***					
		Seoul virus	SEOV	R22	Vero E6-Lyon	NCPV

^a^NCPV, National Collection of Pathogenic Viruses (Porton Down, United Kingdom).

BNI, Bernhard-Nocht-Institute for Tropical Medicine (Hamburg, Germany). IP ASCR, Institute of Parasitology—Academy of Sciences of the Czech Republic (Prague, Czech Republic). UNIBO, University of Bologna (Bologna, Italy).

^b^N.A., not available.

The viral titers were measured as follow: SLEV = 8.1×10^9^ PFU/ml, USUV = 1.35×10^9^ PFU/ml, TBEV = 1.66×10^9^ PFU/ml, JPEV = 5.34×10^7^ PFU/ml, WNV NY99 = 1.5×10^10^ PFU/ml, WNV Dakar = 1.61×10^10^ PFU/ml, CCHFV = 9.6×10^8^ PFU/ml, RVFV = 9.92×10^7^ PFU/ml, and SEOV = 4.66×10^7^ PFU/ml.

### Nucleic acid isolation

Prior to extraction, each cell culture supernatant was concentrated from 1 ml to 100 μl using 10-kDa AMICON Ultra centrifugal units (Merck Millipore; Billerica, MA, United States of America) at 4,000 × *g* for 4 min. After concentration, RNA was isolated and extracted on an EZ1 Advanced XL platform (Qiagen; Hilden, Germany). The EZ1 Virus Mini Kit v2.0 (Qiagen) was used, adhering to the manufacturer’s protocol.

### Real-time PCR and LAMP assays

Real-time PCR assays were performed on a ViiA 7 Real-Time PCR System (Applied Biosystems; Carlsbad, CA, United States of America) using the Power SYBR RNA-to-Ct One-Step kit (Thermofisher Scientific; Bremen, Germany). Reverse transcription was performed at 48°C for 30 min, and samples were subjected to 40 cycles of PCR amplification (95°C for 15 s and 55°C for 1 min) for flaviviruses. The same conditions were used for the members of the *Bunyaviridae* family, except that 52°C was used for the second step of the cycles, instead of 55°C. Amplification was performed in a reaction volume of 50 μl, and amplification products were detected using SYBR Green staining. Due to higher concentrations for the *Flaviviridae*, 3 μl from the initial solution was used as a template instead of 5 μl for CCHFV, RVFV, and SEOV. A final concentration of 0.2 μM was used for both the forward and reverse primers for each reaction. The melting curves were done with temperatures ranging from 55°C to 95°C with a ramp rate of 0.05°C/s. LAMP assays were performed on a 7500 Fast Real-Time PCR System (Applied Biosystems; Carlsbad, CA, United States of America). Isothermal MMX (OptiGene; Horsham, United Kingdom) was used at a 1× concentration in a 12-μl reaction volume. Primers were used at the following concentrations: F3 and B3, 0.2 μM; FIP and BIP, 2 μM; and loopF and loopB, 1 μM.

## Results

### Workflow concept

**Fig 1:**
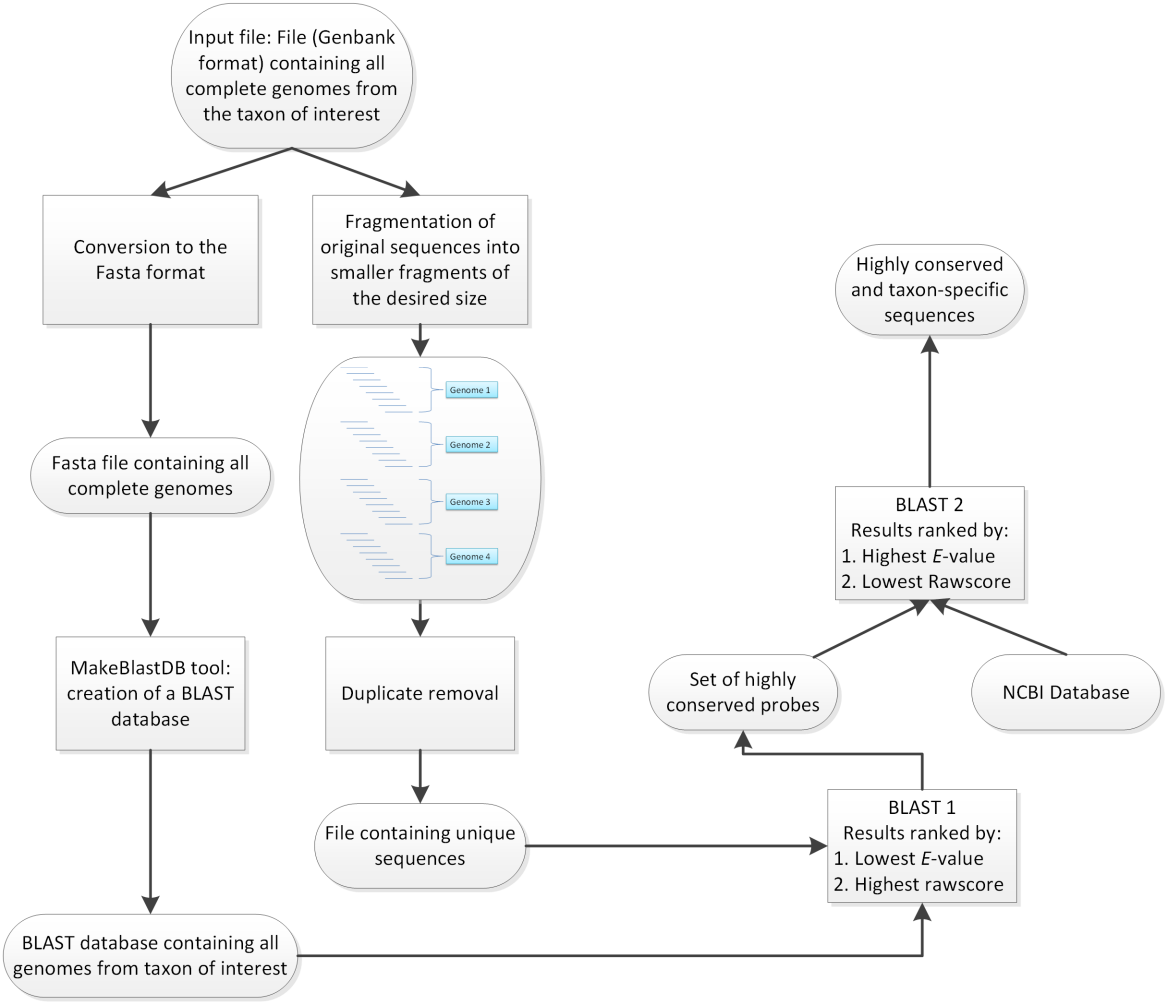
Analysis workflow. Input sequences were processed through a “dual-BLASTn” pipeline in order to select for the most conserved and at the same time specific molecular markers.

The presented approach consisted of two consecutive BLASTn steps to assess the degree of conservation of a sequence among a taxon of interest and to test for its specificity toward closely related organisms, as detailed in Fig 1.

Preprocessing of the whole genomic sequences used as input was carried out in two steps. Genomic samples were first fragmented to 400 bp. Because consecutive fragments shared an overlap of 390 bp, they allowed accurate representation of the various genomic regions for the next processing steps. Two additional filtering steps were used to remove sequences showing suboptimal thermodynamic parameters from this pool of organism-specific fragments (OSFs). The first filter selected only fragments with a GC content of 30–70%, and the second filter checked the remaining fragments for homopolymers or repeated regions, which are generally considered inappropriate targets for molecular assays. In parallel, genomic sequences in GenBank format were converted to Fasta format and further converted into an organism-specific database (OSD) using the appropriate tool provided within the NCBI software suite. Subsequently, the first BLASTn step was carried out to select the HCFs among the taxon of interest. In order to perform this action, OSFs were compared to the OSD. The scores resulting from this analysis, including the total amount of hits in the OSD, *E*-values and bitscores, were retrieved in order to assess the degree of conservation of each OSF in the taxon of interest. Moreover, OSFs were ranked by decreasing number of hits, decreasing sum of bitscores, and increasing sum of *E*-values. A subset (*n* = 100) of the fragments with the best scores was selected for further analysis.

The second part of this workflow aimed to assess the specificity of the subset of HCFs toward the organism of interest, thus providing information on potential cross-reactions with close relatives. This step consisted of an additional BLASTn step against the NCBI’s nt database. In contrast to the ranking system from the previous step, HCFs were ranked by increasing number of hits, increasing sums of bitscores, and decreasing *E*-values, thus enabling ranking to be carried out in accordance with the complete database. Hence, this step allowed us to assess the specificity of each of HCF and served as an assessment of the potential for cross-reactions when using the selected HCFs as targets for molecular assays.

### Genetic diversity among the tested viruses

The consensus sequences from 10 and 60 segment L complete sequences from the CCHFV were generated in order to assess whether using different numbers of sequences could influence the selection of a target for identification assays. For the same reason, two consensus sequences from 10 and 153 complete JPEV genomes were also generated. The results of these alignments are reported in Table 2. The consensus generated from 60 CCHFV sequences had 871 additional ambiguities when compared with the consensus generated from 10 CCHFV sequences. This represents approximately 16% of the overall length of the consensus (5,372 bp). On the other hand, the consensus generated from 153 JPEV sequences had only 48 additional ambiguities when compared with the consensus generated with 10 JPEV sequences, suggesting that only 0.44% of the genome (10,980 bp) represented variable sites.

**Table 2:** Ambiguity-based comparison of consensus sequences generated using various amounts of Crimean-Congo hemorrhagic fever virus (CCHFV) or Japanese encephalitis virus (JPEV) genomes.

	Consensus CCHFV	Consensus JPEV	Consensus CCHFV	Consensus JPEV	Variation CCHFV	Variation JPEV
Sequences	10	10	60	153	N.A.^a^	N.A.
Length (bp)	5,370	10,979	5,372	10,980	2	1
GC (%):	38.18	46.33	30.49	46.45	-7.68	-0.11
A (%):	28.29	24.98	23.59	25.17	-4.70	-0.19
C (%):	18.90	20.13	14.99	20.15	-3.92	-0.02
G (%):	19.27	26.20	15.51	26.30	-3.77	-0.10
T (%):	22.09	17.68	17.09	17.81	-5.00	-0.14
Y (%):	5.51	5.16	11.58	5.11	6.07	0.05
W (%):	0.73	0.44	1.73	0.40	1.00	0.04
V (%):	0.02	0.00	0.24	0.00	0.22	0.00
S (%):	0.04	0.30	0.50	0.26	0.47	0.05
R (%):	4.41	3.96	10.67	3.73	6.25	0.23
N (%):	0.02	0.15	0.58	0.16	0.56	-0.02
M (%):	0.34	0.57	1.62	0.53	1.28	0.05
K (%):	0.24	0.43	0.60	0.36	0.35	0.06
H (%):	0.04	0.00	0.73	0.01	0.69	-0.01
D (%):	0.06	0.00	0.35	0.00	0.30	0.00
B (%):	0.06	0.00	0.24	0.00	0.19	0.00

^a^N.A., not applicable.

### Workflow output

While using configuration 1, it was not possible to align all 608 complete WNV genome sequences with the ClustalW algorithm or the MUSCLE algorithm [36]. Using our workflow allowed us to select candidate molecular markers from different numbers of complete genome sequences, from as few as six sequences for USUV to as many as 608 sequences for WNV. Selected molecular markers were used to generate real-time PCR primer sets for the detection of viruses from both the *Bunyaviridae* and *Flaviviridae* families (Table 3). Because of the lack of published LAMP assays and to demonstrate that the molecular markers selected using this workflow were multipurpose, we used the HCFs for USUV and SLEV to design LAMP primer sets (Table 4).

**Table 3:** List of selected targets and real-time PCR primer pairs designed for different viral species employed in this study.

Species	Target	Forward primer (5’–3’)	Reverse primer (5’–3’)	Sequence numb.	Size (bp)
JPEV	NSP 5	GGTACTACTGGGGCGAATGG	CCAAAAGGGGTGGTGTCAGT	153	342
SLEV	PreMP	ACAAGACTGACGCTCAAAGC	GGATTGCGCAAAACCCAGTT	8	352
TBEV	NSP 5	ACAGCTAAACTTGCCTGGCT	ACGGTTTTTCCACTGCTCCA	42	348
USUV	NSP 5	TCATGGAGCGCTTGGAAGTT	CAGGTCCGATATGGGTGGTC	6	343
WNV	NSP 1	ACCAGAACTCGCCAACAACA	TCTCAAGGATTCCATCGCCC	608	341
CCHFV	Seg.^a^ L	GCATCTCTGAAGTAACTGAAACAACA	GTTGAGATAGCACCGAGTTTCTTTAG	41	154
	Seg. M	AGAAACAAGCTTATCAATTGAGGCAC	TGTCCTTTCTTCCAGCTTCATAATTG	60	175
	Seg. S	GATGAGATGAACAAGTGGTTTGAAGA	GTAGATGGAATCCTTTTGTGCATCAT	65	159
SEOV	Seg. L	GTCTCACTTAGTACGAGTAAGGTTGA	AATTTTTGTCAGACATGCCTATACCG	7	178
	Seg. M	CCTTGCAACAATTGATTCTTTTCAAT	ACAAGGATTCTCAGCCAAATTTTCAA	18	160
	Seg. S	GAAGAAATCCAGAGAGAAATCAGTGC	ATTTTTGATTGTATTGAAGCTGCGAC	19	161
RVFV	Seg. L	ATGATGAATGACGGGTTTGATCATTT	AACCTCATACTTAGCGAGTTTAGTCA	86	150
	Seg. M	GGCCCTTAGAGTTTTTAACTGTATCG	GGGCTCTCAATGAAAGAAAAGCTATT	91	192
	Seg. S	AACAATCATTTTCTTGGCATCCTTCT	ATAATGGACAACTATCAAGAGCTTGC	141	180

^a^Seg., segment;

WNV, West Nile virus; SLEV, St. Louis encephalitis virus; JPEV, Japanese encephalitis virus; USUV, Usutu virus; TBEV, Tick-borne encephalitis virus; SEOV, Seoul virus; CCHFV, Crimean-Congo hemorrhagic fever virus; RVFV, Rift Valley fever virus.

**Table 4:** List of LAMP primer sets designed for Usutu virus (USUV) and St. Louis encephalitis virus (SLEV).

Species	Primer^a^	Primer sequence (5’–3’)	Input sequences
SLEV	F3	GAGCACTTGATGTGGGAG	8
	B3	CAATGATTGCCGAATCGC	
	FIP	CTTCCATCCGTAATCCAACTCATCCTGACTTGTCAGTTGTAGTGC	
	BIP	AACACATTTGTTGTTGATGGACCCGAGTGAACACCATGCCAA	
	LoopF	CCAGCTTCTTCAGGCGTC	
	LoopB	CAAGGAGTGTCCAACAGCA	
USUV	F3	GCTGCCAATGAATACGGA	6
	B3	TAGTGGAGGGTAGCCAGA	
	FIP	GTGAGAACCACTGTGCTCCCTACCCTCCATGAACGCTT	
	BIP	TCAGAATACATCACAACATCTCTGGCGTAGGTTGAACAAAGACCCA	
	LoopF	GGTCGCAAATCCAATGCC	
	LoopB	TTCAATAAGCGCTCAGGC	

^a^F3 and B3, forward outer and reverse outer primers for LAMP, respectively;

FIP and BIP, inner LAMP primers; LoopF and LoopR, forward and reverse loop primers.

**Fig 2:**
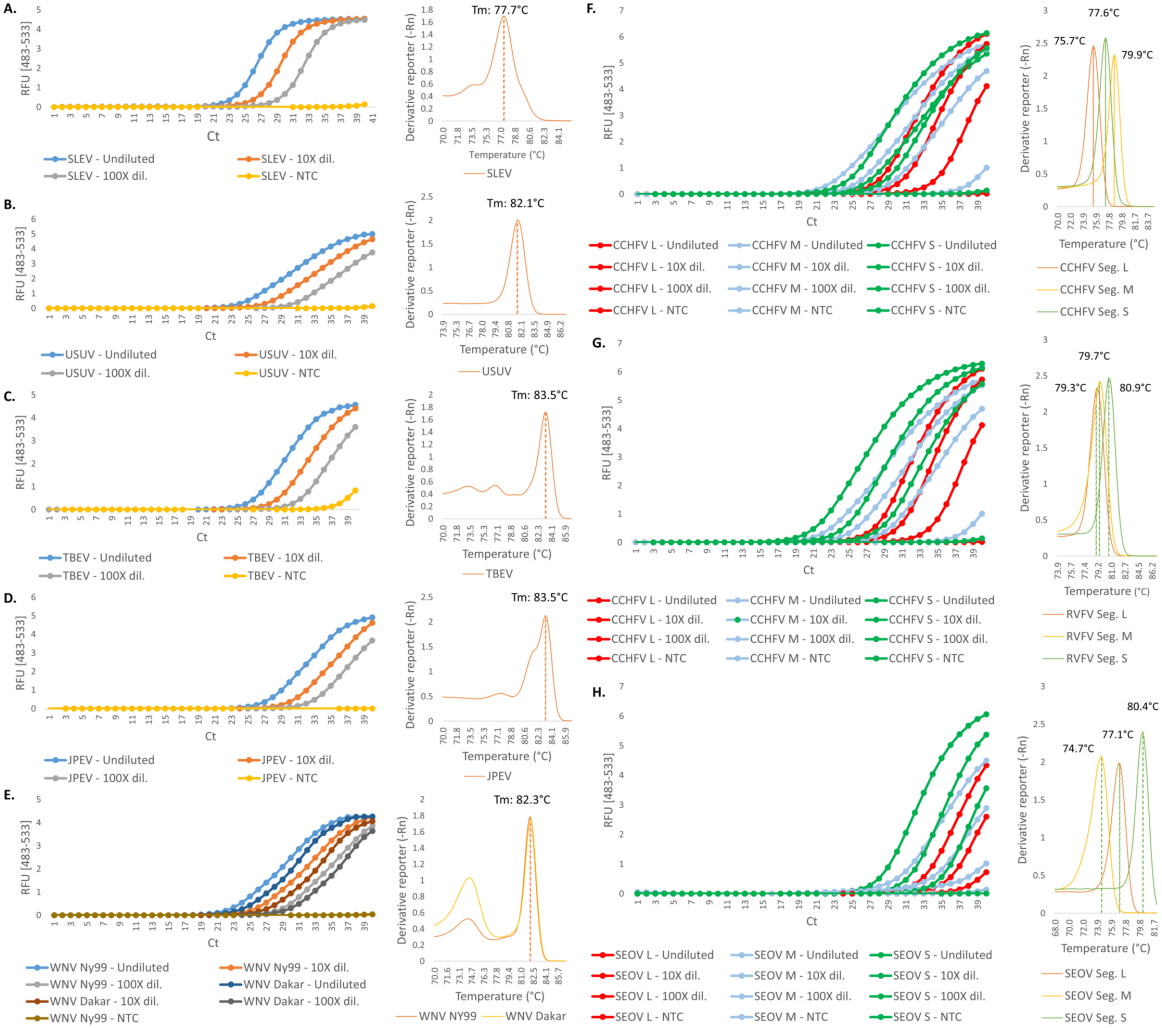
Real-time PCR assays of members from the *Flavivirida*e and *Bunyaviridae* families. Amplification and melting curves for five different flaviviruses species are shown. Each sample was tested undiluted, with a 10-fold dilution and with a 100-fold dilution. (A) St. Louis encephalitis virus (SLEV). (B) Usutu virus (USUV). (C) Tick-borne encephalitis virus (TBEV). (D) Japanese encephalitis virus (JPEV). (E) West Nile virus (WNV; 2 strains, NY99 and Dakar). The right half of the panel shows the amplification and melting curves of the different genomic segments of the members from the *Bunyaviridae* family tested in this study. (F) Crimean-Congo hemorrhagic fever virus (CCHFV). (G) Rift Valley fever virus (RVFV). (H) Seoul virus (SEOV). NTC, no template control; RFU, relative fluorescence units; C_t_, cycle threshold; Dil., dilution; Seg., Segment.

The selected primer pairs were tested against a panel of virus species, including two WNV (NY99 and Dakar) strains, as shown in Fig 2. CCHFV was amplified with an average between the different genomics segments of 21.9 cycles, RVFV with an average of 23 cycles, and SEOV a C_*t*_ value average of 27.8. SLEV, WNV NY99, USUV, and WNV Dakar reached the threshold between 23 and 26 cycles (23.8, 24.1, 25.3, and 25.4, respectively). TBEV and JPEV were amplified within 27.8 and 28.1 cycles, respectively. The efficiency of the reactions was measured between 82% (RVFV Segment M) at the lowest and 141% (JPEV) at the highest. The efficiency of 11 of the other 13 reactions was comprised between 90% and 110% except for TBEV (115%) and CCHFV Segment S (86%).

**Fig 3:**
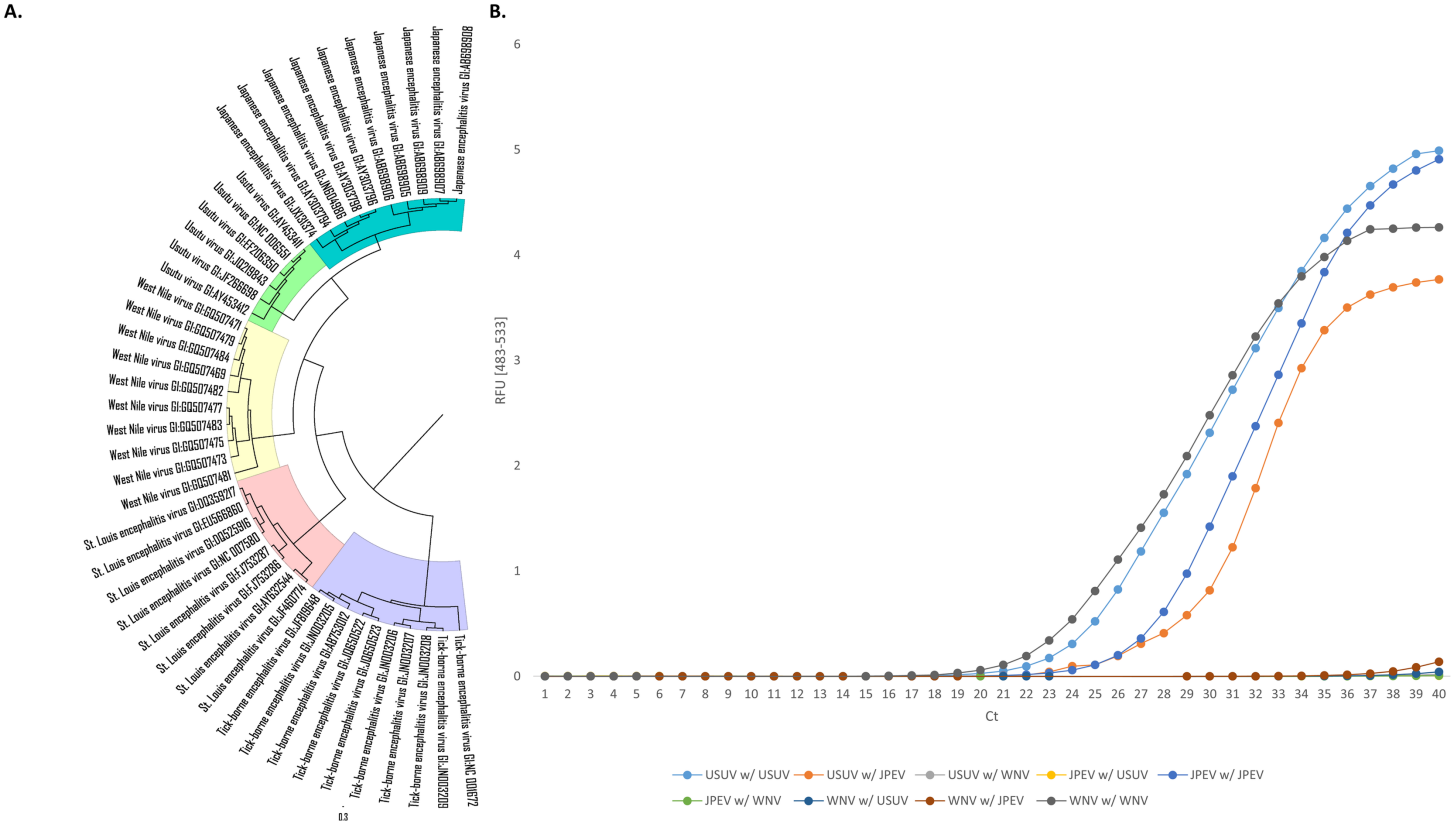
Testing cross-reactions between a set of close relatives from the *Flaviviridae* family. West Nile virus (WNV), Japanese encephalitis virus (JPEV), and Usutu virus (USUV) were tested. (A) Phylogenetic analysis of a subset of 6–10 sequences from members of the *Flaviviridae* family. (B) Real-time amplification of viruses with master mixes containing different primer pairs. RFU, relative fluorescence units; C_*t*_, cycle threshold.

A phylogenetic tree of the *Flaviviridae* family was generated, as shown in Fig 3, in order to test for cross-reactivity between the closest relatives, namely JPEV, USUV, and WNV. As previously shown, fragments of all three species were amplified using the corresponding primer sets at 28.1 cycles for JPEV, 25.3 cycles for USUV, and 24.1 cycles for WNV (NY99).

There was no cross-amplification when mixing the JPEV template with the primer pairs selected for USUV and WNV. Similarly, for WNV, amplification occurred only with the corresponding WNV primers and not with the USUV or JPEV primer pairs. However, while there was no amplification with the USUV template and WNV primers, there was amplification when using JPEV primers around cycle 29.

In order to make sure that this cross-reaction does not involve the selection of the target regions but rather the selection of the primer pairs designed to amplify this region, we sequenced the amplicons from the three relevant reactions, namely (i) the JPEV template amplified with JPEV primers; (ii) the USUV template amplified with JPEV primers and (iii) the USUV template amplified with USUV primers (S1 Supporting Information). The obtained sequences were compared to the NCBI database and the USUV template amplified with the USUV primers showed 61.8% identity with JPEV genomic sequences and 70.3% identity when using the primer pair selected for JPEV.

**Fig 4:**
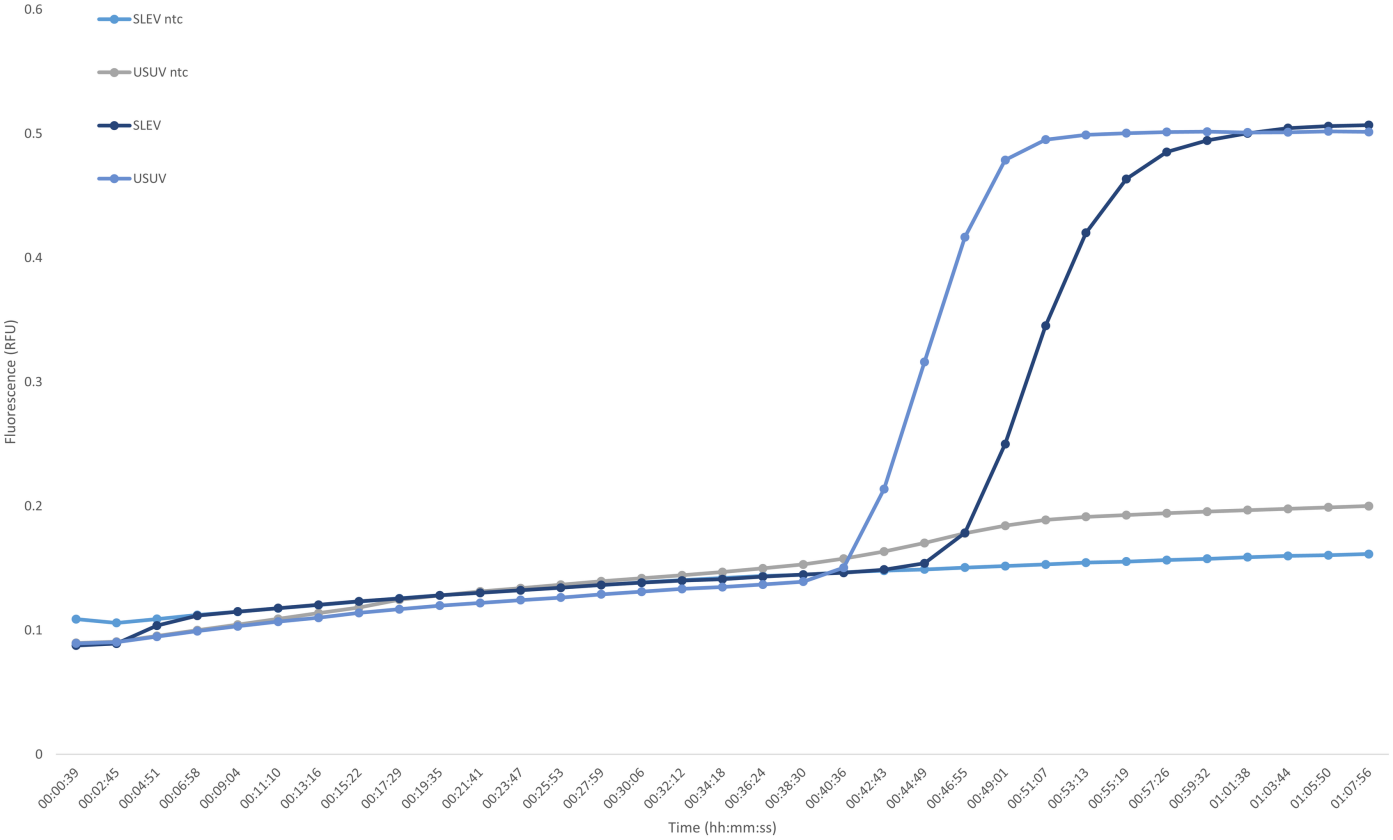
Loop-mediated isothermal amplification of Usutu virus (USUV) and St. Louis encephalitis virus (SLEV). NTC, no template control; RFU, relative fluorescence units; C_*t*_, cycle threshold.

Both USUV and SLEV were successfully amplified with corresponding LAMP assay primers. Amplification occurred after 46 min for SLEV RNA, including the reverse transcription step. The LAMP primer set selected for USUV successfully amplified the template within 40 min, also including the reverse transcription step (Fig 4). The included controls excluded the formation of primer dimers, which is likely to happen due to the nested nature of LAMP assays.

## Discussion

We developed and evaluated a bioinformatics workflow to find species diagnostic markers that readily addresses the high intra-species genetic diversity of viruses and takes into consideration the potential for cross-reactivity between close relatives. These are two key issues that complicate the design of diagnostic molecular assays [21, 37]. Our workflow allowed for rapid selection of highly conserved and specific genomic fragments among the investigated viruses, while considering up to several hundred complete genomic sequences.

With the advent of next-generation sequencing, an increasing number of sequences have been, and continue to be, made publicly available [25, 38]. Although this has greatly improved our knowledge of the dynamics of viral populations, the massive amount of data available also renders bioinformatics analysis more complex. In the case of CCHFV, for example, the difference in the consensus sequences between analyses utilizing 10 and 60 genomic sequences was 17.39%, which is a challenge for selecting an appropriate target for a molecular assay. For JPEV, the amount of variable sites was much lower, only representing 0.45% of the complete genome; nonetheless, 50 additional ambiguities were observed throughout the whole consensus. Yet, even such a small difference might still negatively influence the performance of a molecular assay by affecting the thermodynamic parameters of the reaction, particularly the primer annealing step.

Aligning a few genomic sequences is usually straightforward with widely available bioinformatics tools [39, 40]. In the case of organisms that have not been as thoroughly sequenced, alignment may not be an issue at all because all available variants may simply be included in the alignment; thus, the overall genetic diversity is considered. In the case of extensively sequenced organisms, however, the issue of “masked” diversity might rise, since only a subset of all the available sequences will be selected for the alignment and finally only a subset of the genetic diversity is taken into account for the design of the molecular assay. By using reproducible computing scores, including bitscores, *E*-values, and the number of “hits” in a database, the workflow also removed the potential bias that could be introduced by manual selection of an adequately amplified region by the user. This workflow allowed us to select highly specific molecular markers in less than an hour for all tested viruses using the more powerful configuration 2. In order to assess the impact of the hardware, we ran the workflow with a single species on both configurations. While the task could be successfully completed on both computer platforms, we noted a drop in the time requirement of approximately 30% from configuration 1 to 2. This drop in performance was thought to be due to the well-optimized parallelization capacity of the BLASTn algorithm. Therefore, we expected that the overall runtime could be reduced by increasing the number of CPU cores and providing sufficient RAM. In future studies, we will examine the importance of this feature in terms of increased sequencing capacity and the increased resulting genomic data generated every year [38]. The performance of this workflow will also allow re-running the analyses when new sequences for a given species of interest become available. This would facilitate identification of shifts in the viral population and could reveal whether previously selected molecular markers are still valid (*i*.*e*., to keep the molecular assay up-to-date and to have it further refined as new data become available). In specific cases, if enough sequences are available, this workflow could also be utilized to generate strain-specific molecular markers. Having strain-specific assays, particularly in the case of neglected tropical diseases, could be a great asset when tracking/investigating transmission events and risk factors, in resource-constrained settings [41, 42]. This workflow also has the advantage of manual design, and hence, it can be entirely customized to the needs of the user. In fact, the output from the workflow only depended on the input sequences, and the user should be able to select, for example, only geographically related strains to design a “geographically specific” assay in order to quickly demonstrate whether outbreaks are caused by a new or re-emerging pathogen [43].

All molecular markers that were selected with the workflow could be used as inputs for primer design. Real-time PCR assays were all performed successfully, from the single amplification target selected for the flaviviruses to the three regions selected for each genomic fragment of the members from the *Bunyaviridae* family. Similarly, the same markers selected for USUV and SLEV were successfully used to design LAMP primer sets, and the corresponding LAMP assays performed well. These assays confirmed that the first BLASTn step of this workflow functioned well for selecting highly conserved regions among a pool of species-specific fragments.

The results generated within this study offer a preliminary overview of the assays sensitivity and specificity. However, additional experiments would be required to optimize these assays, especially concerning the efficiency of reaction. In general, the melting curves show a high specificity, except for WNV for which some primer-dimers seem to be forming. Regarding the suboptimal efficiencies, one lead to optimize could be to remove either inhibitors (especially in the case of JPEV and TBEV, which show an increased reaction efficiency), test various primer concentrations as well as a range of more adapted, reaction-specific, PCR conditions.

In order to further improve this workflow, we added a second BLASTn step to assess the degree of sharing of highly conserved species-specific fragments in a general database also containing genomic data from close relatives. The tested cross-reactions showed that the primers selected for WNV and USUV were specific for those species, whereas the JPEV primers cross-reacted with the USUV template, but not with the WNV template. In order to determine whether this cross-reaction occurred because of the primers or poor selection of the molecular markers, we used Sanger sequencing to sequence the amplicons from the two USUV reactions (both with USUV and JPEV primers) and the JPEV reaction (with the JPEV primers). Sequencing revealed that the amplified regions (*i*.*e*., the selected molecular markers) were highly specific to their corresponding species. An online BLASTn of the JPEV primers against USUV sequences showed that the forward primer had nine nucleotides matching the USUV virus at the 3′ end and 19 common nucleotides on the reverse primer (only one mismatch, data not shown). This issue highlights two additional controls that should be performed using this workflow after selecting the target regions, namely (i) an additional online BLASTn control of the primer selected by the various software programs, be it for real-time PCR or LAMP assays, and (ii) since cross-reactions are difficult to predict, the designed assay should be tested with a gradient PCR first to ensure that the thermodynamic parameters of the reaction are optimal. However, sequencing of the amplification product is still considered the ‘gold’ standard for validating the molecular assay and ensuring high specificity of the assay.

In conclusion, the workflow presented here for developing diagnostic markers for viral species identification provides a promising approach as it addresses the recurrent issue of bioinformatics analysis of large amounts of sequencing data, which is expected to be an even greater challenge as publicly available data are rapidly increasing. This workflow removes user-introduced bias by being solely based on well-established computing scores (bitscore, *E*-value, and number of hits). Hence, our workflow addresses two issues encountered in the manual design of a molecular assay, as it takes into account the complete genetic diversity of a species, and provides timely information on potential cross-reactions to close relatives. We speculate that our workflow is applicable to a variety of DNA-based assays, and hence, it should theoretically work for higher organisms, such as bacteria or parasites, facilitating the selection of future diagnostic markers.

## Supporting information

S1 Table Accession numbers. (XLSX)

S1 Supporting Information Sequenced amplicons. (FASTA)
